# Hydroxylated fullerene promotes fracture healing in ovariectomized combined type 2 diabetic mice

**DOI:** 10.1186/s13018-025-05997-5

**Published:** 2025-06-19

**Authors:** Yunpeng Hu, Linjie Feng, Jie Li, Fuyuan Cao, Xiaoli Hou, Wei Chen, Yimeng Zhang, Lei Xing, Jingyuan Gao, Liu Zhang, Ye Liu, Faming Tian

**Affiliations:** 1 School of Public Health, North China University of Science and Technology, Tangshan, Hebei 063000 PR China; 2 Department of Orthopedic Surgery, The Second Hospital of Tangshan, Tangshan, Hebei 063000 China; 3 Department of Orthopedic Surgery, The 982th Hospital of Joint Logistic Force of Chinese People’s Liberation Army, Tangshan, Hebei 063000 China; 4 Beijing National Research Center for Molecular Sciences, Key Laboratory of Molecular Nanostructure and Nanotechnology, Institute of Chemistry, Chinese Academy of Sciences, Beijing, 100080 China; 5 Department of Orthopedic Surgery, Hebei Medical University, Shijiazhuang, Hebei 050023 China; 6 Medical Department, The First Hospital of Hebei Medical University, Shijiazhuang, 050023 China; 7 Department of Rheumatology and Immunology, Affiliated Hospital of North China, University of Science and Technology, Tangshan, Hebei 063000 China; 8 Department of Anaesthesiology, Beijing Obstetrics and Gynecology Hospital, Capital Medical University, Beijing, 100069 China

## Abstract

**Background:**

The aim of this investigation was to assess the interventional role of hydroxylated fullerene in this model by histological, imaging, and biomechanical means in order to inform the treatment of fracture healing in a population of menopausal women with type 2 diabetes mellitus (T2DM).

**Methods:**

Sixty-four female 8-week-old C57BL/6 mice were randomly divided into four groups: fracture (F), OVX + fracture (OF), T2DM + OVX + fracture (DOF), and hydroxylated fullerene-treated DOF (DOFF). A closed fracture was established in the right tibia of each mouse, which was performed 8 weeks after undergoing ovariectomy (OVX) in the OF, DOF, and DOFF groups. Hydroxylated fullerene (5 mg/kg, every two days) was administered intraperitoneally to the DOFF group starting the day after fracture. The right tibias were collected at 7 and 28 days post-fracture.

**Results:**

The systemic administration of hydroxylated fullerene significantly increased the microstructural parameters of the callus (including bone volume fraction [BV/TV] and connectivity density [Conn.D]), promoted BMP-2 expression and inhibited TNF-α expression in the callus at 7 days post-fracture, and increased the expression of type I collagen (COL1) and osteocalcin (OCN) in the callus at 28 days post-fracture.

**Conclusion:**

Hydroxylated fullerene may improve fracture healing in this diabetic mouse model by reducing the inflammatory response and promoting osteogenesis. These results suggest that hydroxylated fullerene may act as a therapeutic agent for fracture healing.

## Background

Estrogen deficiency is a cause of age-related osteoporosis, resulting in significant bone loss and reduced bone strength in postmenopausal women. This ultimately increases the risk of fracture [1]. Poor bone quality due to osteoporosis also undermines the successful surgical treatment of these fractures and reduces the stability of the bone fixation structure [2]. Pre-clinical studies have shown that both early and late phases of fracture healing are impeded in ovariectomy (OVX)-induced animal models of osteoporosis [1].

Type 2 diabetes mellitus (T2DM), a frequent co-occurring condition in obesity, is characterized by elevated blood sugar levels due to insulin resistance as well as impaired function of the insulin-producing pancreatic beta cells [3]. Chronic hyperglycemia can also impact the metabolic homeostasis of bone by promoting the synthesis of advanced glycation end products (AGEs) and the release of reactive oxygen species (ROS) [4]. Over the past few decades, a series of animal and human studies have consistently shown that T2DM adversely affects fracture healing by disrupting the balance of bone homeostasis, doubling the fracture healing time and increasing the risk of delayed healing and bone non-union in diabetic patients compared to non-diabetic patients [5, 6].

T2DM and osteoporosis are common and complex conditions that often occur together. Epidemiological studies have established a higher occurrence of T2DM in postmenopausal women compared to premenopausal women [7]. The combination of estrogen deficiency and T2DM worsens impaired fracture healing [8, 9]. With the increasing incidence of T2DM, there has been an accompanying increase in postmenopausal osteoporosis comorbid with diabetes [10]. In this population, developing effective treatment and interventions for fracture healing is a medical challenge that should be prioritized.

While current therapies, including pharmacological agents and physical interventions, have been applied in fracture repair, their therapeutic efficacy is frequently constrained within the complex pathological microenvironment of osteoporosis. For instance, bisphosphonates demonstrate limited capacity to improve callus quality in diabetic models, while the bone-healing potential of parathyroid hormone remains contentious in diabetic conditions [11]. Regarding physical modalities, although intermittent pneumatic compression (IPC) and low-intensity pulsed ultrasound (LIPUS) facilitate fracture healing through enhanced local perfusion and mechanical stimulation, their effectiveness in diabetic bone defect models remains inconclusive [12, 13]. Given these therapeutic limitations, the current investigation centers on elucidating the distinctive therapeutic potential of fullerene (a carbon-based nanomaterial with approximate 0.7 nm diameter) in the context of diabetic osteoporotic fracture healing. Recent studies have found that fullerene and its derivatives have significant biological properties. In vitro studies have revealed that fullerenes exhibit dual therapeutic effects by attenuating inflammatory responses in the tumor microenvironment [14] and ameliorating type 2 diabetes mellitus (T2DM) through reactive oxygen species (ROS) scavenging [15]. Furthermore, fullerenes directly stimulate osteoblast proliferation and differentiation [16, 17] while suppressing osteoclastogenesis by inhibiting bone marrow macrophage (BMM) differentiation [18]. Animal studies confirm their bone-protective effects against both LPS-induced and estrogen deficiency-related bone loss [19, 20]. However, the role of fullerenes in osteoporotic fractures caused by T2DM comorbid with estrogen deficiency remains unknown. The primary objective of this study was to evaluate the therapeutic potential of hydroxylated fullerene (a water-soluble fullerene derivative) in fracture healing using a closed tibia fracture model. We employed ovariectomized mice with comorbid T2DM, induced through a well-established protocol combining streptozotocin (STZ) administration and high-fat diet to recapitulate human T2DM pathophysiology. Through histological, imaging, and biomechanical analyses, we aimed to assess its interventional effects, thereby informing potential treatments for fracture healing in postmenopausal women with T2DM.

## Methods

### Animals and treatment

**Fig. 1:**
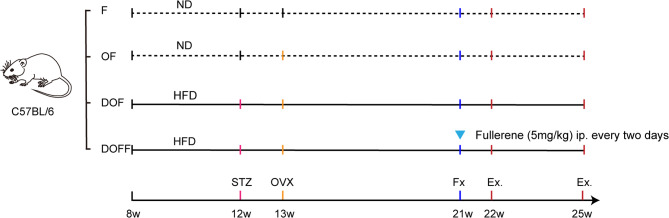
Experimental protocol. ND: normal diet. HFD: high-fat diet. STZ: streptozotocin injection. OVX: ovariectomized. Fx: fracture. Ex.: excute

All experiments were approved by the Institutional Animal Care and Use Committee of the North China University of Science and Technology. Sixty-four eight-week-old female C57/BL6 mice (VitalRiver Experimental Animal Technical Co., Ltd., Beijing, China) were used in this study. The mice were bred in our SPF animal facilities, and every cage contained two to four mice under standard conditions with a 14-hour light/10-hour dark cycle. All of the mice were randomly and equally divided into two groups, one of which was provided with regular chow (catalog number: 1025, HFK Bioscience CO. LTD, Beijing) and the other of which was fed with high-fat chow (catalog number: H10141, HFK Bioscience Co. Ltd., Beijing). These feeding patterns were maintained until the end of the experiment. After 4 weeks of dietary conditioning, a single low dose of STZ (S8050, Solarbio) contained in 10 mM citrate buffer was administered intraperitoneally to the high-fat chow-fed mice, while the vehicle alone was administered to the regular chow-fed mice. One week after administering the mice with STZ by injection, the diabetes model was considered successfully established in mice whose tail blood glucose reading exceeded 13.9 mmol/L [21, 22]. Mice with substandard blood glucose levels were excluded. Blood glucose was thereafter monitored weekly until the end of the experiment. Of the remaining animals, the normal-fed mice were randomly divided into a control group (F, *n* = 18) and an ovariectomized (OVX) group (OF, *n* = 18); the high-fat-fed mice were divided into a T2DM comorbid with OVX group (DOF, *n* = 15) and a T2DM comorbid with OVX hydroxylated fullerene-treated group (DOFF, *n* = 15). Bilateral OVX was performed at 1 week after STZ injection on mice in the OF, DOF, and DOFF groups, while only adipose tissue near the ovaries was removed bilaterally in the F group [23]. Tibial fracture modeling was performed on all mice 8 weeks after OVX surgery. A third of each group was randomly selected to be euthanized by CO_2_ at 7 d post-fracture; the remaining mice were euthanized at 28 d post-fracture. The serum and fractured tibia were retained (Fig. 1). Samples for the three-point bending test were stored at -80℃.

### Tibia fracture model and hydroxylated fullerene intervention

Eight weeks after undergoing OVX, the tibial fracture model was established as previously described [24]. Briefly, a 5-mm incision was made in the medial skin of the knee before a 30 G sterile needle(0.3 × 25 mm, MINANK, Suzhou, China) was inserted through the medullary cavity from the medial tibial plateau. A closed tibial fracture was then established using a homemade three-point bending device. X-rays were taken to identify whether the modeling was successful. Excess fixation pins were subtracted, and the incision was closed. Hydroxylated fullerene (purity > 99.5%, Beijing Fullcan Co., Ltd.) were dispersed in sterile purified water (2 mmol/L) and filtered (0.22 μm) [25]. Solution stability was confirmed by dynamic light scattering prior to each administration. Starting the day after fracture surgery, hydroxylated fullerene were injected intraperitoneally at a concentration of 5 mg/kg every two days into the DOFF group of mice. The control groups (including DOF group) received equal volumes of sterile purified water using identical injection procedures and schedules.

### Micro-CT analysis

After the tibial fracture samples were fixed in 4% neutral formaldehyde for 48 h, the intramedullary fixation pins and excess soft tissues surrounding the bone callus were removed. At 28 d post-fracture, the calluses were examined by high-resolution micro-CT (SkyScan1176; Bruker, Billerica, MA, USA)). Measurements were conducted at 50 keV excitation energy with 270 µA current, providing 8.96580 μm³ spatial resolution. CTAn (v1.20.8.0, Bruker) and CTVoX (v2.3.3.0) were used for analysis and 3D visualization. The region of interest included the 2 mm space above and below the fracture line [26]. The following microstructural parameters were analyzed: bone mineral density (BMD), mineralized volume fraction (BV/TV), connectivity density (Conn. D), trabecular thickness (TB.Th), trabecular number (TB.N), trabecular separation (TB.Sp), and trabecular thickness (TB.Th) [27].

### Histology

Formaldehyde-fixed samples were decalcified using 10% EDTA (pH 7.2–7.4) for over 8 weeks until a 1-ml needle could penetrate the cortical bone without resistance. Samples were then embedded in paraffin and sectioned at 5 μm. The sections were dewaxed and hydrated in xylene and a graded series of alcohol to water for histologic staining. Staining was performed to quantitatively evaluate bony callus formation within the fracture sites using the Safranin O and Fast Green Staining kit from Solarbio (catalog #G1371). Slides with fracture callus sections were independently scored in a blinded fashion by 3 trained orthopedic pathologists.

### TRAP stain

Fracture callus sections were prepared following a standardized processing protocol established previously (see Sect. “Histology”). To evaluate the extent of osteoclast-mediated bone resorption within the fracture healing area, samples collected at 7 days and 28 days post-fracture were stained using the Tartrate-resistant acid phosphatase (TRAP) staining kit (catalog #G1492) from Solarbio, following the manufacturer’s recommended procedures [28]. The region of interest (ROI) was defined as the area adjacent to the fracture line at 200× magnification. TRAP-positive cells near the fracture site were quantified using Image-Pro Plus 6.0 software, and osteoclast density (N/mm^2^) was subsequently calculated.

### Immunohistochemistry

Fracture healing involves a multifaceted pathological process comprising overlapping biological phases. Distinct cells and biological factors assume significant roles in each phase of the healing process [29–31]. Based on this characterization, we detected the expression of TNF-α and BMP-2 at 7 days post-fracture, as well as type I collagen (COL1) and osteocalcin (OCN) at 28 days post-fracture in the calluses of different groups. In brief, paraffin sections were dewaxed and then repaired by 0.05% trypsin and the inactivation of endogenous peroxidases with hydrogen peroxide (H_2_O_2_) at room temperature for 15 min. Immunohistochemistry staining was performed using the primary antibodies anti-TNF-α (1:150; BA0131, Boster) and anti-BMP-2 (1:200; ARG57829, Arigo) for samples taken at 7 d post-fracture, while antibodies anti-COL-1 (1:150; ARG54605, Arigo) and anti-OCN (1:150; PB1008, Boster) were used for samples taken at 28 d post-fracture to evaluate their expression in the callus. Relative factors expressed in bone callus were quantified by measuring mean optical density (IOD), as previously described [32].

### Biomechanics

The universal testing machine (MMT-250NV-10; Shimadzu, Kyoto, Japan) was used to evaluate the mechanical properties. Tibia specimens collected at 28 days post-fracture were positioned patella-side up on a 3-point bending apparatus with two 6 mm-separated supports. The tibia callus was mechanically loaded at 1.0 mm/min velocity until callus failure occurred [33]. Maximum load (N) and energy to failure (mJ) were calculated from the load-displacement curves.

### Serum assays

Serum harvested at 28d post-fracture was used for further analysis. Serum concentrations of OCN (RX202908M, Ruixin biotech) and the receptor for advanced glycation end products (RAGE) (RX202582M, Ruixin biotech) were determined using enzyme-linked immunosorbent assay (ELISA) kits according to the manufacturer’s instructions [27].

### Data analysis and statistics

All data have been presented as the mean ± SD. Based on the homogeneity results of variance analysis with Levene’s test, the groups were compared using either one-way ANOVA followed by Tukey’s post-hoc tests or the Kruskal-Wallis H test followed by Dunn’s post-hoc test. The level of statistical significance was accepted at *P*-values < 0.05. Statistical analysis data were processed using SPSS software (SPSS v20.0; IBM, Armonk, New York, USA).

## Results

### Blood glucose levels

**Fig. 2:**
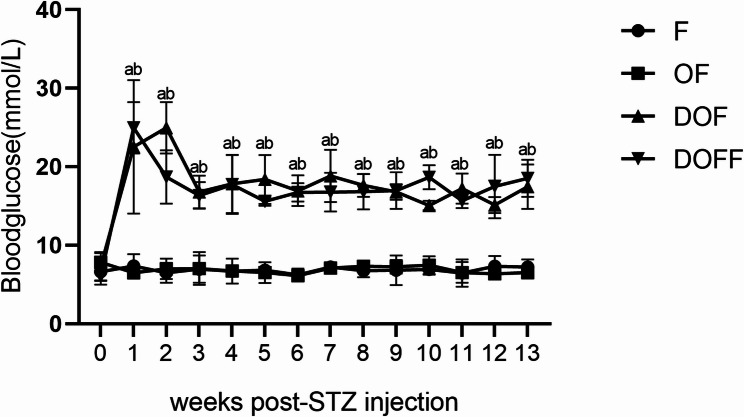
Blood glucose concentrations after streptozotocin (STZ) injection. Data are presented as the mean ± SD. ^a^*P*<0.05 DOF group vs. F group. ^b^*P*<0.05 DOFF group vs. F group

One week post-STZ injection, blood glucose increased significantly in DOF and DOFF groups vs. F and OF groups. (Fig. 2).

### Micro-CT

**Fig. 3:**
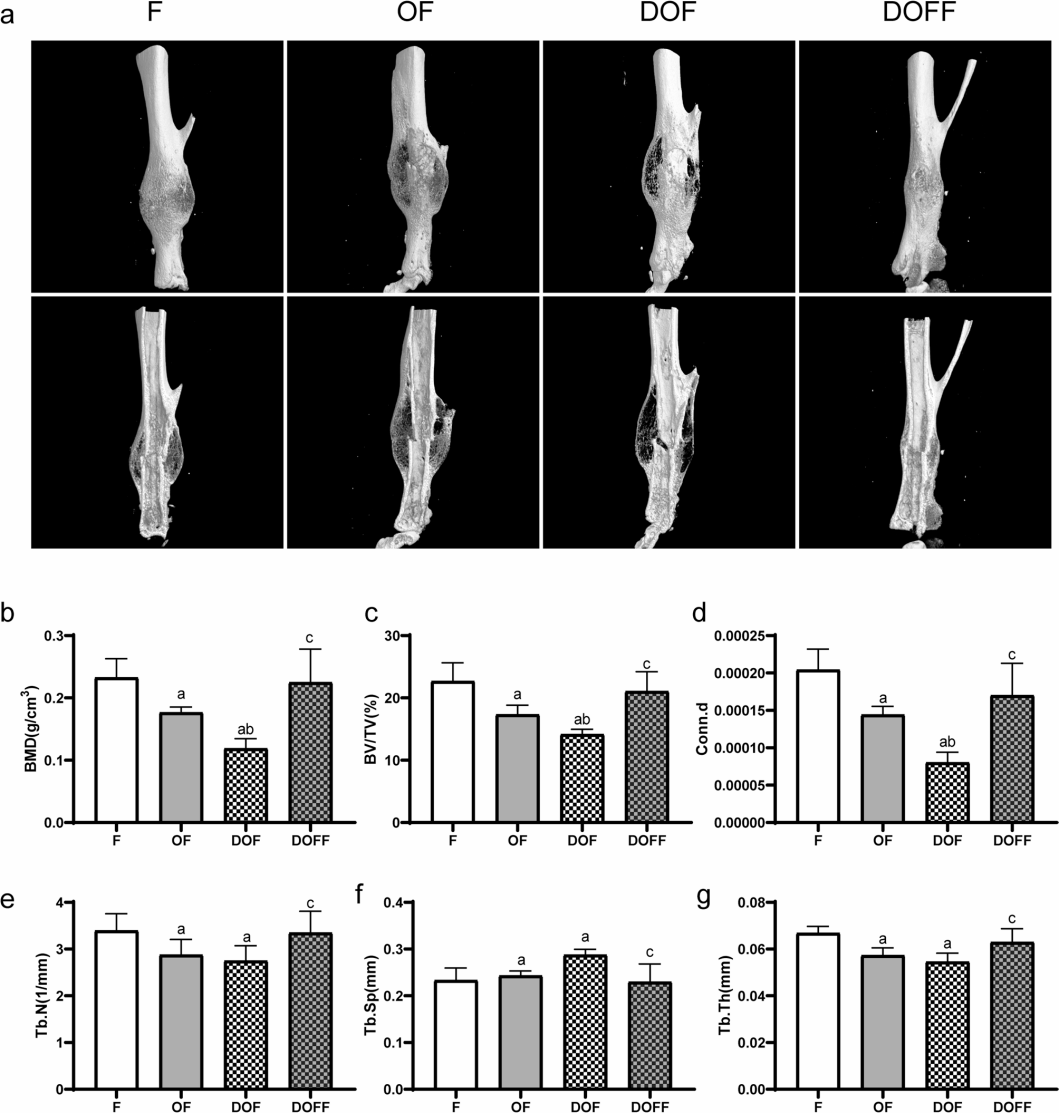
Micro-CT analysis at 28 d post-fracture. (**a**) Micro-CT 3D reconstruction images. (**b-g**) Bone mineral density (BMD), bone volume/total volume (BV/TV), connectivity density (Conn.D), trabecular number (Tb.N), trabecular separation (Tb.Sp), trabecular thickness (Tb.Th) in the callus for each group. Data are presented as the mean ± SD. ^a^*P*<0.05 vs. F group, ^b^*P*<0.05 vs. OF group, ^c^*P*<0.05 vs. DOF group

The micro-CT 3D reconstruction image is shown in Fig. 3a. At 28 days after fracture, the values of BMD, BV/TV, and Conn.d at the bone callus site were significantly lower in the DOF group than in the F, OF, and DOFF groups (all *P* < 0.05). The Tb.N andTb. Th values at the bone callus site were significantly lower in the OF and DOF groups than in the F group; conversely, they were considerably higher in the DOFF group than in the DOF group (all *P* < 0.05). In comparison with group F, the Tb.Sp was significantly higher in the OF and DOF groups; conversely, it was considerably lower in the DOFF group compared to the DOF group (all *P* < 0.05) (Fig. 3b-g).

### Histology

**Fig. 4:**
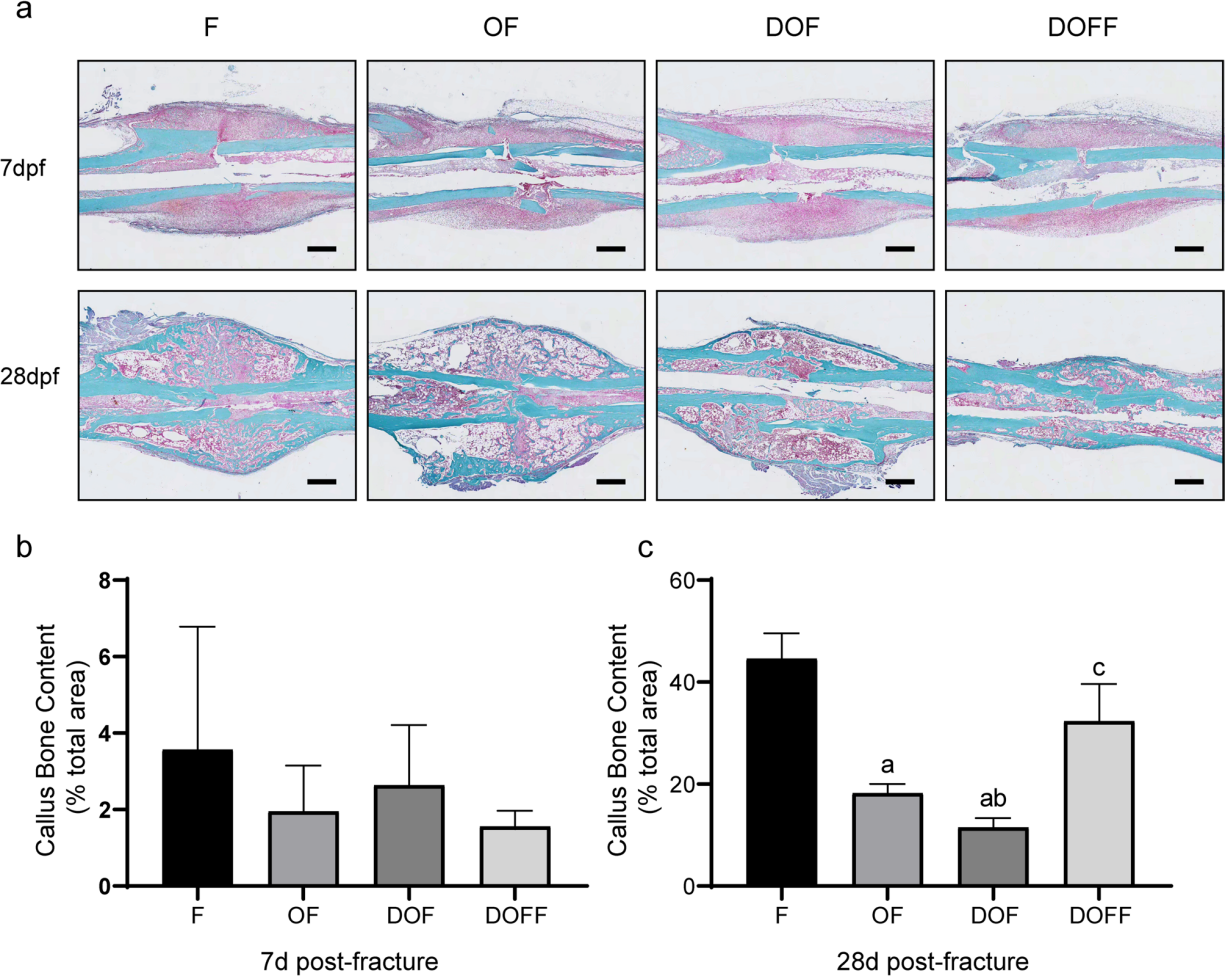
(**a**) Histological results showing the sagittal sections of callus from 7 and 28 d post-fracture. Bar = 500 μm. (**b-c**) Bone content of the callus at different time points. Data are presented as the mean ± SD. ^a^*P*<0.05 vs. F group, ^b^*P*<0.05 vs. OF group, ^c^*P*<0.05 vs. DOF group

Safranin O/Fast Green staining quantified bony callus content(Fig. 4a). Consistent with the micro-CT results, the callus bone content at 28 d post-fracture was significantly lower in the DOF group than in the F, OF, and DOF groups (all *P* < 0.05). However, no significant differences existed in callus bone content between groups at 7 days post-fracture. (Fig. 4bc).

### TRAP

**Fig. 5:**
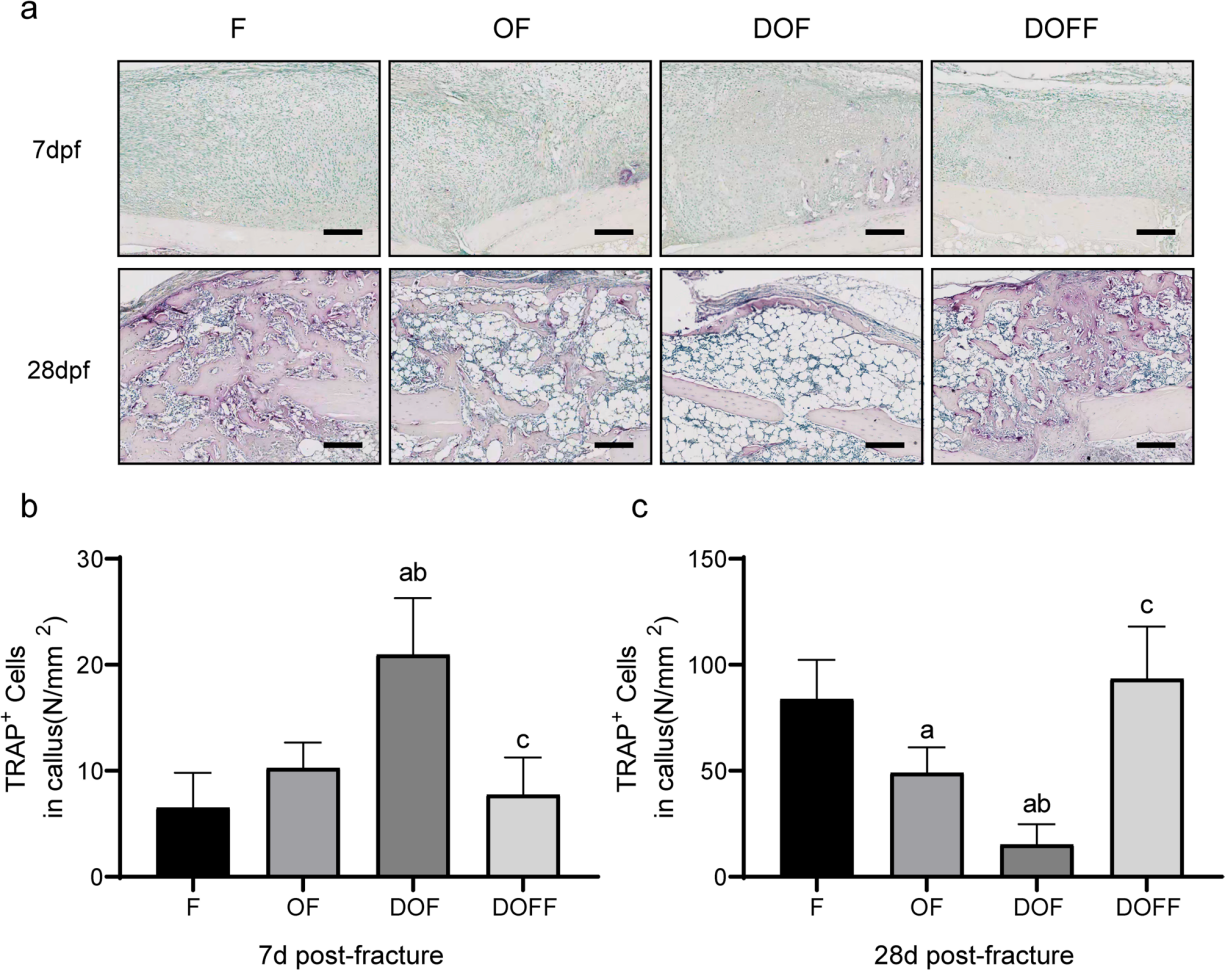
TRAP-stained sections with the quantitation of osteoclast density (N/mm^2^) in the calluses. Bar = 200 μm. Data are presented as the mean ± SD. ^a^*P*<0.05 vs. F group, ^b^*P*<0.05 vs. OF group, ^c^*P*<0.05 vs. DOF group

Osteoclast numbers in the callus at 7 d and 28 d after fracture were evaluated by TRAP staining (Fig. 5a). In the callus specimens at 28 days after fracture, the number of osteoclasts in the OF group was significantly lower than that in the F group, while the number of osteoclasts in the DOF group was markedly lower than that in the F, OF, and DOFF groups. At day 7 after fracture, the number of osteoclasts in the DOF group was significantly higher than in the other groups (all *P* < 0.05) (Fig. 5bc).

### Immunohistochemistry

**Fig. 6:**
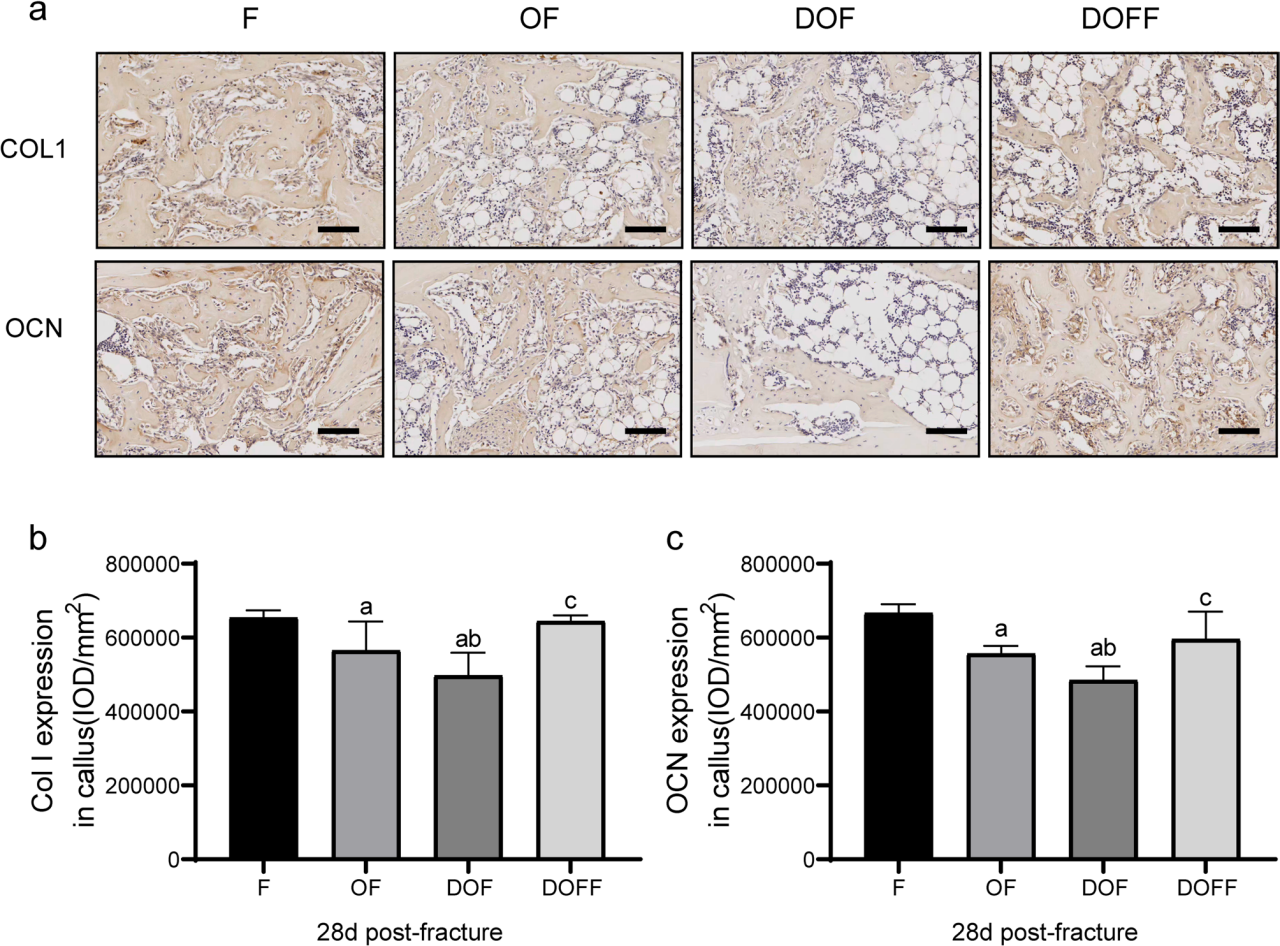
(**a**) Immunohistochemical staining results of type I collagen (COL1) and osteocalcin (OCN) in the callus for each group at 28 d post-fracture. (**b-c**) Quantitation of collagen-I and OCN expression in the calluses. Bar = 100 μm. Data are presented as the mean ± SD. ^a^*P*<0.05 vs. F group, ^b^*P*<0.05 vs. OF group, ^c^*P*<0.05 vs. DOF group

**Fig. 7:**
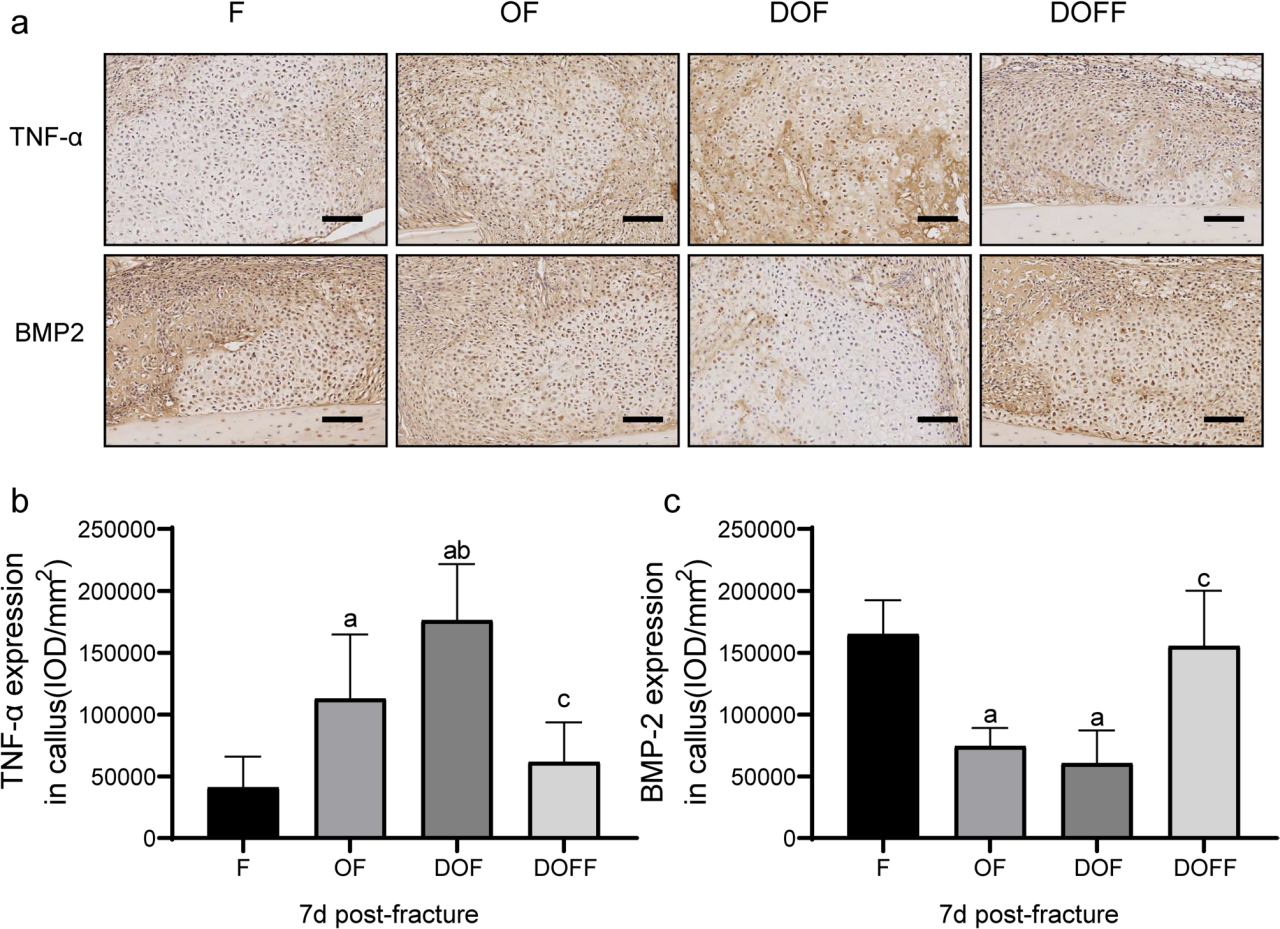
(**a**) Immunohistochemical staining results of TNF-α and BMP-2 in the callus for each group at 7 d post-fracture. (**b-c**) Quantitation of TNF-α and BMP-2 expression in the calluses. Bar = 100 μm. Data are presented as the mean ± SD. ^a^*P*<0.05 vs. F group, ^b^*P*<0.05 vs. OF group, ^c^*P*<0.05 vs. DOF group

At 28 d post-fracture, the callus expression levels of COL I and OCN (Fig. 6a) in the DOF group were significantly lower than those in the F, OF, and DOFF groups (all *P* < 0.05) (Fig. 6 bc). At 7 d post-fracture, the expression of TNF-α in the callus was significantly higher in the DOF group than in the F, OF, and DOFF groups. While significantly lower levels of BMP-2 in the DOF group than in the F and DOFF groups (all *P* < 0.05), the DOF and OF groups showed no difference (Fig. 7a-c).

**Fig. 8:**
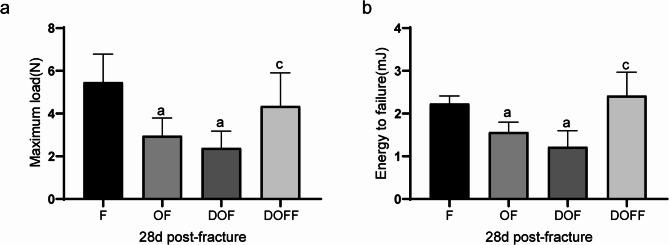
Biomechanical test results and serum analysis. (**a**) Maximum load. (**b**) Energy to failure. Data are presented as the mean ± SD. ^a^*P*<0.05 vs. F group, ^b^*P*<0.05 vs. OF group, ^c^*P*<0.05 vs. DOF group

### Biomechanics

The three-point bending test was conducted to determine the strength of the callus 28 days after fracture (Fig. 8ab). Compared to the F and DOFF groups, the maximum load and the energy to failure were significantly lower in the DOF group (all *P* < 0.05). There was no significant difference between the DOF and OF groups regarding the above two parameters.

### Serum assays

**Fig. 9:**
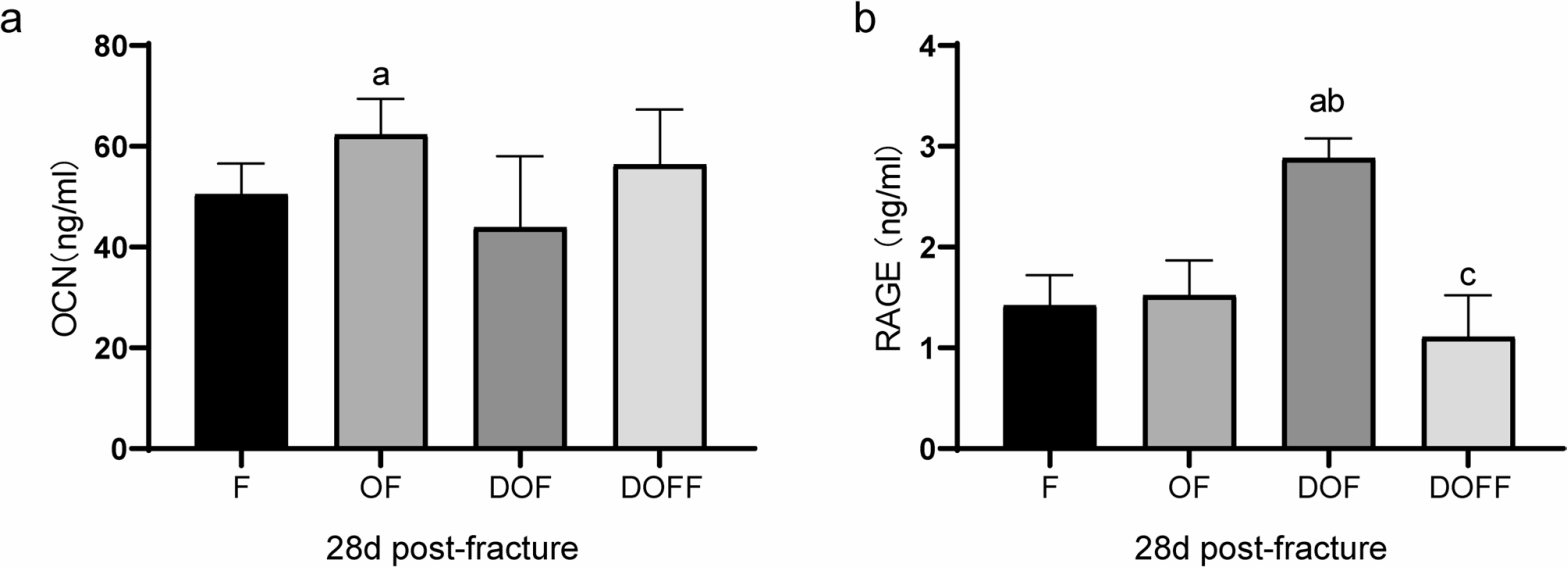
Serum analysis. (**a**) Osteocalcin (OCN) and (**b**) receptor for advanced glycation end products (RAGE) concentrations in the serum. Data are presented as the mean ± SD. ^a^*P*<0.05 vs. F group, ^b^*P*<0.05 vs. OF group, ^c^*P*<0.05 vs. DOF group

Serum osteocalcin (OCN) increased in OF vs. F groups (*P* < 0.05) but did not differ between DOF and other groups (Fig. 9a). Serum RAGE was elevated in DOF compared to F, OF and DOF groups (all *P* < 0.05) (Fig. 9d).

## Discussion

This study elucidated two findings. First, it determined whether abnormally elevated blood glucose levels can exacerbate the delay in fracture healing and reduce the quality of fracture healing in estrogen-deficient mice. This was done by administering STZ to induce T2DM and performing OVX to model estrogen deficiency in mice, in which a fracture model was then established. The findings showed that mice in the DOF group exhibited more immature callus tissue compared to the OF group. Second, we determined the positive effect of the systemic administration of hydroxylated fullerene on fracture healing in the above models. Based on imaging, histology, immunohistochemistry, and biomechanical assays, we found that hydroxylated fullerene intervention promoted the fracture healing process and increased the callus strength in a mouse model of T2DM combined with estrogen deficiency.

Estrogen is a key hormone required for bone homeostasis. Its deficiency causes the skeleton to exhibit a high bone turnover phenotype, whereby bone resorption and bone formation increase simultaneously, but bone formation exceeds bone resorption [34]. It is well established [35–37] that fracture healing is significantly inhibited in OVX-induced osteoporotic mice. In our study, both histologic and micro-CT results showed a significant decrease in the bone mineral density alongside a deterioration in the microstructural parameters of newly generated bone in the OF group at 28 days post-fracture, as well as delayed bone remodeling, which is consistent with previous studies [38, 39]. Consistent with the histologic and micro-CT results, the compressive capacity of the callus was significantly reduced 28 days after fracture in estrogen-deficient mice. Immunohistochemical results showed that at 28 days post-fracture, the significant reductions observed in type I collagen (COL1) and osteocalcin (OCN) expression in the callus of the OF group also indicated a reduction in callus quality and osteogenic capacity during bone repair. BMP-2 is an important osteogenic peptide that is required to initiate fracture repair [40] and promote the resorption and ossification of the cartilaginous callus [41]. There is evidence that both estrogen deficiency and T2DM inhibit the expression of BMP-2 in the callus [36, 42]. In our study, we found that estrogen deficiency led to a decrease in BMP-2 expression in the callus during the early stages of fracture healing. This may be due to the lack of estrogen binding to the ERα response element in the BMP-2 promoter, resulting in decreased BMP-2 transcription [36, 43]. TNF-α is a major mediator of the inflammatory process during the initial stages of fracture healing, which then returns to its baseline level during the ensuing repair process [44, 45]. However, abnormally elevated levels of TNF-α for an extended period due to estrogen deficiency may interfere with fracture healing [46]. Chow et al. found that OVX downregulated TNF-α expression in the calluses of rats during the early stage of fracture healing, which was a pattern in contrast to its elevation in the serum [47]. However, in our study on mice, the expression of TNF-α in the OF group was significantly elevated at 7 days after fracture. This disparity may be due to the different model animals used and the fact that the fracture healing cycle in mice is shorter than in rats, such that at the time point of 7 days in mice, the expression of COL II in the callus of the tibial fracture was already significantly elevated [31], and this healing process is not part of the early inflammatory phase.

Extensive research confirms that T2DM alone negatively impacts bone metabolism through various pathways, including oxidative stress, AGE deposition, enhanced marrow adipogenesis, and visceral fat-derived inflammatory factors and adipokines. These alterations ultimately compromise osteoblast, osteoclast, and osteocyte function via direct and indirect effects [48, 49]. Both human and animal models of T2DM exhibit impaired fracture healing [50]. It has been demonstrated that during fracture healing in rats, T2DM combined with estrogen deficiency greatly delays the fracture healing process, promotes the apoptosis of chondrocytes and osteoblasts, and affects bone remodeling [8, 51]. Similar to previous studies, our study employed a mouse model to find that 28 days after fracture, the DOF group had worse microstructural parameters and lower bone mass in the callus compared to the OF group. However, our biomechanical results did not reflect the observed histological and radiological changes, and a study by Aeimlapa et al. also found that T2DM did not significantly exacerbate the reduction in maximum loading and energy to failure in the intact femur due to estrogen deficiency [52]. In our study, T2DM exacerbated the estrogen deficiency-induced reduction of OCN and COL1 expression in the callus. This is similar to the results of another study of fracture healing in rats [51], which suggested that high blood glucose levels and estrogen deficiency have a combined effect on the fracture healing process by decreasing bone formation and increasing bone resorption activity. Contrary to our expectations, our results showed that T2DM combined with estrogen deficiency did not exacerbate the expression of BMP-2. We found that TNF-α was upregulated in T2DM patients, suggesting that TNF-α plays an important regulatory role in the delayed healing of T2DM fractures, which is in accordance with a study by Sun et al. [53, 54]. The relatively higher TNF-α expression impaired the subsequent bone repair process. In our study, T2DM exacerbated the expression of TNF-α in the osteoclasts of estrogen-deficient mice. A similar phenomenon was observed in a survey by Raehtz et al. [8].

Fullerene and its derivative have been shown to enhance bone formation by enhancing osteogenic differentiation-related genes and matrix mineralization [55]. They have also been reported to promote new bone formation in cranial defects in rats [56]. However, the effect of fullerene application on fracture healing in T2DM combined with estrogen deficiency in an animal model has not yet been reported. In our results, hydroxylated fullerene significantly promoted the formation of new bone in the callus. Based on the results of our histological analysis conducted on samples taken 28 days post-fracture, the following was found: first, bone volume was increased in the DOFF group compared to the DOF group; second, the DOFF group underwent the process of callus remodeling; and third, the bony callus was more mature and compact. The micro-CT results also showed that the calluses in the DOFF group had higher bone volume as well as better microstructural morphology. Biomechanical tests indicated that the intervention with hydroxylated fullerene resulted in a significant increase in the strength of the callus compared to the DOFF group. In summary, the above evidence suggests that the application of hydroxylated fullerene significantly improved the microstructure of the callus in mice with T2DM combined with estrogen deficiency and facilitated the process of fracture healing. At 28 days post-fracture, the results of the immunohistochemical analysis showed a significant upregulation of COL I and OCN expression following hydroxylated fullerene intervention. This observation is consistent with previous findings from histological, imaging, and biomechanical studies, all of which collectively suggest that hydroxylated fullerene exert a promotive influence on fracture healing in this particular model. Substantial evidence has demonstrated that fullerene and its derivative enhance adhesion, proliferation, and differentiation of both osteoblast-like cells (MG-63 cell line) and human-deprived osteoblasts, although direct mechanistic evidence remains insufficient [57, 58]. Our experimental results revealed a significant upregulation of BMP-2 expression in fracture callus at 7 days post-fracture following hydroxylated fullerene treatment. This suggests that hydroxylated fullerene-mediated elevation of BMP-2 levels may promote the expression of osteoblast-specific genes (e.g., OCN, COL1) through the following mechanisms: (1) directly activating the BMP/Smad pathway (via Smad1/5/8 phosphorylation) [59] to enhance osteogenic differentiation of mesenchymal stem cells (MSCs), and (2) indirectly functioning through activation of Runx2 and Osterix [60]. These synergistic mechanisms collectively contribute to fracture healing. Additionally, fullerene nanomaterials have been demonstrated to mitigate TNF-α secretion by scavenging excessive ROS or modulating redox homeostasis, thereby suppressing the activation of inflammation-associated signaling pathways such as NF-κB and MAPK/ERK in tumor microenvironment [14, 61]. In our study, TNF-α expression was significantly lower compared to the DOF group, suggesting that hydroxylated fullerene may promote fracture healing in mice with T2DM combined with estrogen deficiency by reducing the inflammatory response at the fracture site [62]. Several studies have demonstrated the promotive effect of TNF-α on osteoclast differentiation [63–65], which may explain the significantly higher number of osteoclasts observed in the DOF group compared to the other groups in the early stages (at 7 days) of fracture healing.

While this study demonstrates the therapeutic potential of hydroxylated fullerene, several limitations should be acknowledged. First, while our combined T2DM and ovariectomy model effectively mimics postmenopausal diabetic conditions, the absence of a T2DM-only control group limits our ability to fully dissect the independent effects of hyperglycemia. Second, although we demonstrated fullerene’s modulation of TNF-α and BMP-2 pathways, the precise molecular mechanisms - particularly regarding fullerene’s interaction with key signaling molecules remain to be fully elucidated. Third, our biomechanical assessment was limited to three-point bending tests at a single time point (28 days), without evaluating earlier mechanical properties or crack propagation patterns during healing. Finally, the use of young mice (8-week-old) may not fully replicate age-related bone healing challenges seen in clinical populations.

## Conclusion

In this study, a fracture model in mice with T2DM combined with estrogen deficiency was successfully established. Hydroxylated fullerene may improve fracture healing in this model by reducing inflammatory responses and promoting osteogenesis. Our results suggest that hydroxylated fullerene may act as therapeutic agents for fracture healing, thereby expanding their application potential.

## Data Availability

No datasets were generated or analysed during the current study.

## Abbreviations

OVX: Ovariectomy
T2DM: Type I diabetes mellitus
TRAP: Tartrate-resistant acid phosphatase
Col-I: Type 1 collagen
OCN: Osteocalcin
BMD: Bone mineral density
BV/TV: Bone volume/total volume
Tb.N: Trabecular number
Tb.Sp: Trabecular separation
